# Proteomic Profile of Endometrial Cancer: A Scoping Review

**DOI:** 10.3390/biology13080584

**Published:** 2024-08-01

**Authors:** Beatriz Serambeque, Catarina Mestre, Kristina Hundarova, Carlos Miguel Marto, Bárbara Oliveiros, Ana Rita Gomes, Ricardo Teixo, Ana Sofia Carvalho, Maria Filomena Botelho, Rune Matthiesen, Maria João Carvalho, Mafalda Laranjo

**Affiliations:** 1 Univ Coimbra, Coimbra Institute for Clinical and Biomedical Research (iCBR) Area of Environment Genetics and Oncobiology (CIMAGO), Institute of Biophysics, Faculty of Medicine, 3000-548 Coimbra, Portugal; uc48103@uc.pt (C.M.); cmiguel.marto@uc.pt (C.M.M.); arpgomes@student.uc.pt (A.R.G.); uc2008114703@student.uc.pt (R.T.); mfbotelho@fmed.uc.pt (M.F.B.); mjcarvalho@fmed.uc.pt (M.J.C.); 2 Univ Coimbra, Center for Innovative Biomedicine and Biotechnology (CIBB), 3000-548 Coimbra, Portugal; boliveiros@fmed.uc.pt; 3 Gynecology Service, Department of Gynecology, Obstetrics, Reproduction and Neonatology, Unidade Local de Saúde de Coimbra, 3004-561 Coimbra, Portugal; 11650@ulscoimbra.min-saude.pt; 4 Clinical Academic Centre of Coimbra (CACC), 3004-561 Coimbra, Portugal; 5 Univ Coimbra, Institute of Experimental Pathology, Faculty of Medicine, 3000-548 Coimbra, Portugal; 6 Univ Coimbra, Institute of Integrated Clinical Practice and Laboratory for Evidence-Based Sciences and Precision Dentistry, 3000-075 Coimbra, Portugal; 7 Univ Coimbra, Centre for Mechanical Engineering, Materials and Processes (CEMMPRE), Advanced Production and Intelligent Systems (ARISE), 3030-788 Coimbra, Portugal; 8 Univ Coimbra, Coimbra Institute for Clinical and Biomedical Research (iCBR) Area of Environment Genetics and Oncobiology (CIMAGO) and Laboratory of Biostatistics and Medical Informatics (LBIM), Faculty of Medicine, 3004-531 Coimbra, Portugal; 9 Univ Coimbra, Chemical Engineering and Renewable Resources for Sustainability (CERES), Faculty of Pharmacy, Laboratory of Pharmaceutical Chemistry, 3000-548 Coimbra, Portugal; 10 iNOVA4Health, NOVA Medical School (NMS), Faculdade de Ciências Médicas (FCM), Universidade Nova de Lisboa, 1150-082 Lisboa, Portugal; ana.carvalho@nms.unl.pt (A.S.C.); rune.matthiesen@nms.unl.pt (R.M.); 11 Univ Coimbra, Universitary Clinic of Gynecology, Faculty of Medicine, 3004-561 Coimbra, Portugal

**Keywords:** biomarkers, diagnosis, endometrial cancer, prognosis, proteomics, therapeutics

## Abstract

**Simple Summary:**

Proteomics can be very useful in identifying proteins, which helps find potential markers for diseases. Managing endometrial cancer can be difficult and finding reliable markers can contribute to an early diagnosis, to manage its evolution, and even predict the response to treatment. This paper reviews the current research on the proteins involved in endometrial cancer. Most studies used tissue, serum, and plasma samples and found potential diagnostic and prognostic markers. Eight studies were examined closely, with three showing strong similarities, sharing forty-five proteins. This review also identified the 10 most commonly reported proteins in these studies. While proteomics shows promise in finding diagnostic and prognostic markers for endometrial cancer, there is still a need for more research on new therapeutic targets.

**Abstract:**

Proteomics can be a robust tool in protein identification and regulation, allowing the discovery of potential biomarkers. In clinical practice, the management of endometrial cancer can be challenging. Thus, identifying promising markers could be beneficial, helping both in diagnosis and prognostic stratification, even predicting the response to therapy. Therefore, this manuscript systematically reviews the existing evidence of the proteomic profile of human endometrial cancer. The literature search was conducted via Medline (through PubMed) and the Web of Science. The inclusion criteria were clinical, in vitro, and in vivo original studies reporting proteomic analysis using all types of samples to map the human endometrial cancer proteome. A total of 55 publications were included in this review. Most of the articles carried out a proteomic analysis on endometrial tissue, serum and plasma samples, which enabled the identification of several potential diagnostic and prognostic biomarkers. In addition, eight articles were analyzed regarding the identified proteins, where three studies showed a strong correlation, sharing forty-five proteins. This analysis also allowed the identification of the 10 most frequently reported proteins in these studies: EGFR, PGRMC1, CSE1L, MYDGF, STMN1, CASP3 ANXA2, YBX1, ANXA1, and MYH11. Proteomics-based approaches pointed out potential diagnostic and prognostic candidates for endometrial cancer. However, there is a lack of studies exploring novel therapeutic targets.

## 1. Introduction

Globally, endometrial cancer is the second most incident gynecology malignancy, with a higher incidence in high-income populations [1].

Presently, molecular classification is employed to characterize and stratify endometrial cancers [2]. This method classifies endometrial carcinomas based on four genetic backgrounds: DNA polymerase ε (POLE, ultramutated), microsatellite instability (MSI, hypermutated), and low and high copy number variations, intending to enhance the treatment outcomes according to the tumor molecular signature [3]. 

The standard-of-care treatment for endometrial cancer includes a total hysterectomy with bilateral salpingo-oophorectomy, with or without lymphadenectomy [4]. Surgical staging is a fundamental procedure in managing endometrial cancer [2], with prognostic and therapeutic implications. Currently, the sentinel lymph node (SLN) biopsy has been recommended for staging instead of lymphadenectomy for low-risk endometrial cancers [4]. Moreover, recent findings indicated that SLN biopsy is considered a reliable approach with a higher sensitivity [5].

Tumor heterogeneity encompasses inter- and intra-tumor variability, and both are challenging for disease management [6]. Endometrial cancer displays tumoral heterogeneity associated with their subtypes, molecular characteristics, and microenvironment, increasing the complexity of the prognosis and treatment of the disease [6,7,8]. This heterogeneity also makes it difficult to identify particular cell populations, such as cancer stem cells (CSC), involved in tumorigenesis, prognosis, and therapeutic outcomes of endometrial cancer patients [7]. This population, which represents a minor percentage of cancer cells within a tumor, is considered a fundamental player in intra-tumor heterogeneity [6,9]. These cells are responsible for resistance to conventional therapies, triggering disease development, spreading, and recurrence [9,10], and are a promising therapeutic target [11].

Proteomics can be a powerful tool, providing information about protein identification and expression levels [12], spanning from cells, tissues, and fluids to entire organisms [13]. Mass spectrometry (MS)-based proteomics is broadly used in the biomarker’s discovery phase [14], but also can be used in the application phase by developing targeted MS proteomics assays, such as selected reaction monitoring (SRM), parallel reaction monitoring (PRM), and Sequential Windowed Acquisition of All Theoretical Fragment Ion Mass Spectra (SWATH-MS) [15]. In cancer research, proteomic studies contribute to understanding pathogenesis, providing valuable insights into tumor heterogeneity—one of the most challenging aspects of cancer research. Additionally, they can assist in identifying diagnostic and prognostic biomarkers and new therapeutic targets [12,13]. Moreover, proteomics can offer essential information into cancer-associated signaling pathways, including cancer development, metastatic potential, and drug resistance [12].

**Figure 1:**
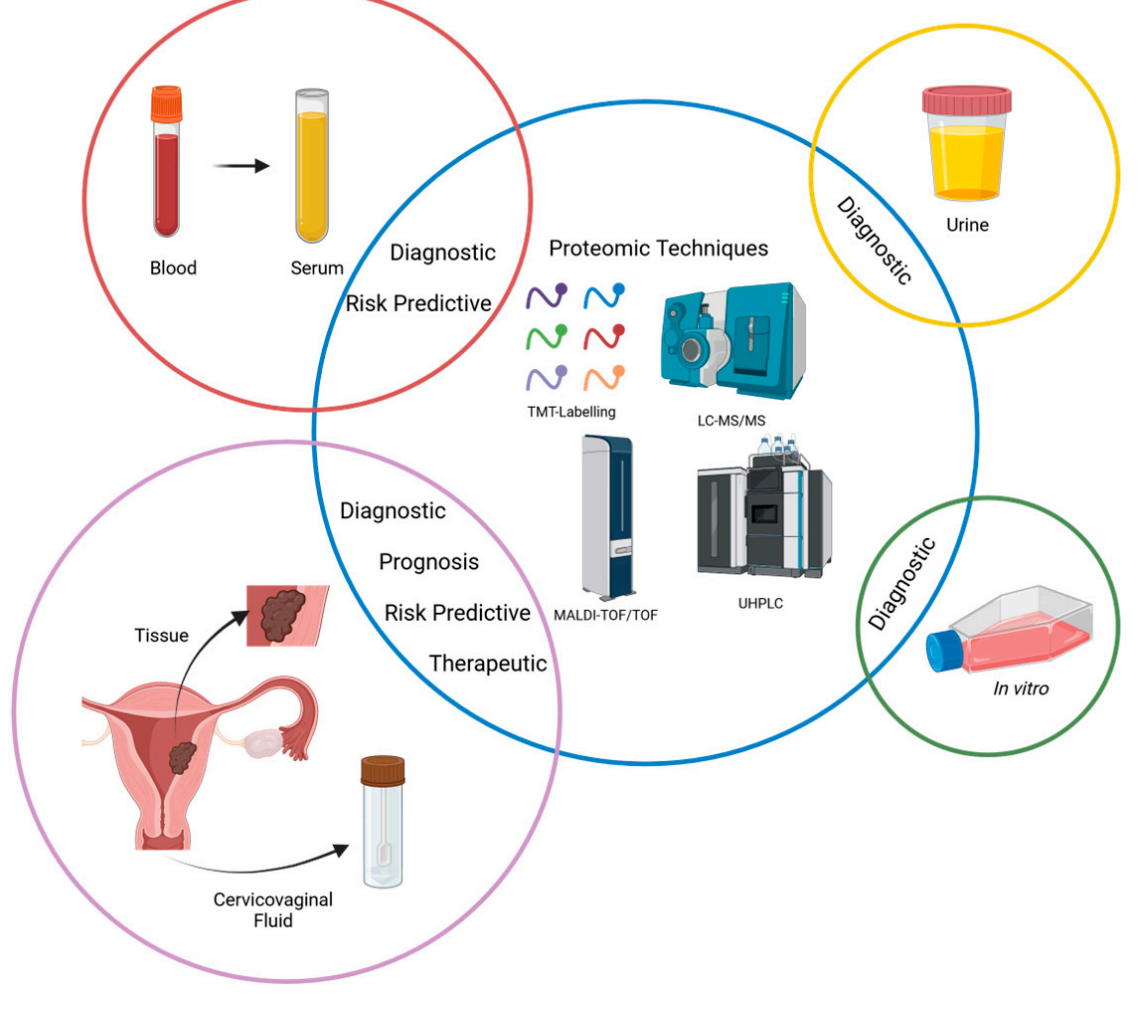
Different proteomic-based approaches applied to several types of samples led to the identification of potential biomarkers for human endometrial cancer. Created with BioRender.com.

The applicability of omics-based approaches has been extensively addressed in gynecologic disorders, including endometrial cancer. However, to the best of our knowledge, a comprehensive description of the endometrial cancer proteome and the identification of possible biomarkers in all types of samples using various proteomic techniques available remains an underexplored topic. Therefore, this study aims to systematically review the proteomic profile of human endometrial cancer, including identifying potential diagnostic, disease progression, and prognostic biomarkers and therapeutic targets (see Figure 1).

## 2. Materials and Methods

This review was planned and conducted according to the methodological framework proposed by Arksey and O’Malley [16] and the “Preferred Reporting Items for Systematic Reviews and Meta-Analysis, extension for Scoping Reviews (PRISMA-ScR)” guidelines [17]. 

### 2.1. Review Question

A review question was structured according to the population, concept and context (PCC) model [18]: “What is the proteomic profile of human endometrial cancer?” Secondary research questions were also formulated: “What is the potential diagnostic and prognostic impact of human endometrial cancer proteomics?”; and “Can human endometrial cancer proteomics identify possible therapeutic targets?”.

### 2.2. Literature Search

The literature search was performed in the Medline (through PubMed) and Web of Science databases. The PubMed search strategy included (“Endometrium”[Mesh] OR Endometri* OR “Endometrial Neoplasms”[Mesh] OR “Carcinoma, Endometrioid”[Mesh] OR “corpus uteri” OR “uterine corpus”) AND (“Proteomics”[Mesh] OR proteomic* OR “Proteome”[Mesh] OR proteome). In Web of Science, the search strategy was (Endometri* OR “corpus uteri” OR “uterine corpus”) AND (proteomic* OR proteome). The document types selected were Article OR Other OR unspecified OR review article OR clinical trial OR letter OR Early Access OR correction. A language filter was used, and articles in English, Portuguese, Spanish, or French were considered in both searches. No temporal restrictions were applied. The most recent search was carried out on 12 October 2023.

### 2.3. Studies Selection 

Database search results were imported to online EndNote, and duplicates were removed. The results were first screened by title and abstract and later by full text. The eligibility criteria included original studies (clinical, in vitro, in vivo, or ex vivo studies), addressing human endometrial cancer and proteomic analysis, encompassing all types of samples (i.e., tissues, cells, serum, blood, and urine), aiming to determine the proteomic signature of human endometrial cancer. Articles addressing endometrial benign disease, comparisons between normal endometrium and benign diseases, or comparisons between endometrial cancer and other malignancies were excluded. Additionally, articles that reported other omic analyses, articles without identification of proteins, as well as searches in databases were also excluded. 

Moreover, the reference lists of the review articles on the topic were screened to identify additional relevant papers. If they met the inclusion and exclusion criteria, such articles were included as cross-references. 

Three researchers independently screened the articles. Three meetings were held to compare each researcher’s selection and reach a consensus decision. Two additional researchers were consulted if needed. 

### 2.4. Data Extraction

The data were collected using a standardized approach, using pre-defined extraction forms for each sample type. The data collected include the total number of samples used and the number of samples for each study group, the proteomics technique used, the patient’s age (when applicable), the main results obtained, and the methods used to validate the proteomics results (when applicable). The results were summarized in tables, and a narrative description was performed. 

This review follows the “Meta-Analyses extension for Scoping Reviews (PRISMA-ScR) Checklist” review protocol [17].

### 2.5. Analysis of Potential Biomarkers

To assess the consistency of the proteins identified in the included studies, eight articles were compared using the statistical programming language R. A network analysis of identified proteins was performed using the R package “igraph.” Also, the visualization of Venn diagrams was made with the R package “VennDiagram”.

## 3. Endometrial Cancer Proteome

**Figure 2:**
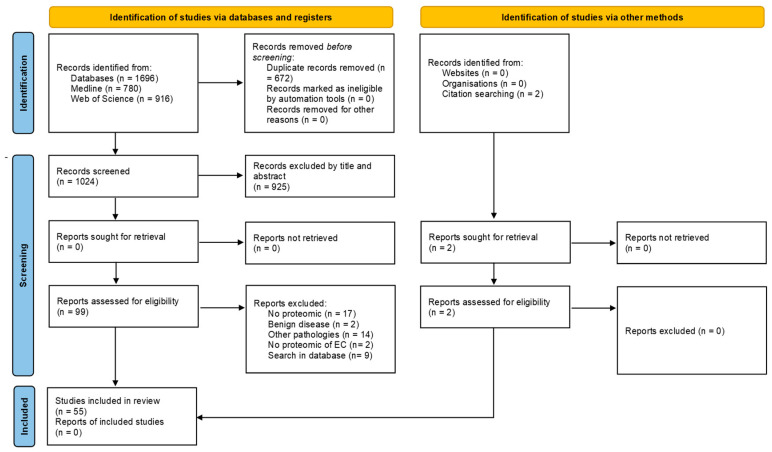
Flow diagram of the selection of evidence sources included and reasons for exclusion.

The search for an endometrial cancer proteomic signature and the discovery of potential biomarkers using proteomic-based methods have been extensively documented across various sample types, including clinical, in vivo, and in vitro. Figure 2 details the articles screened and included and the reasons for exclusion at each phase.

### 3.1. Endometrial Tissue

An endometrial tissue sample can be obtained through a biopsy, a less invasive procedure with good performance in the detection of cancer [19], or in the context of a hysterectomy, the standard treatment for endometrial cancer [4]. Regarding the clinical samples, the use of endometrial tissue, including uterine aspirates, was described in thirty-four papers and detailed in Table 1.

To screen proteins associated with the occurrence and development of endometrial cancer, total tissue extracts of normal endometrium and endometrial cancer were analysed through surface-enhanced laser desorption/ionization time-of-flight mass spectrometry (SELDI-TOF-MS). A set of differentially expressed proteins was found in tumor samples, where CPN10 (HSP10) was identified with an increased expression and indicated as a candidate biomarker for endometrial carcinogenesis [20]. Cancer and paracancerous tissue samples were analysed using a label-free quantification (LFQ) method based on the liquid chromatography and tandem mass spectrometry technique (LC-MS/MS). A total of 3245 proteins were identified, of which 579 were significantly upregulated, and 346 were significantly downregulated, thus accounting for 925 differentially expressed proteins. Seven were selected from this set, given the highest statistical significance: IFIT3, PARP9, SLC34A2, CYB5R1, and PTPN1 were upregulated; and DPT and SLPI were downregulated. Succeeding studies with DPT using quantitative reverse transcription polymerase chain reaction (RT-qPCR) and Western blot (WB) techniques revealed that this protein was significantly downregulated in endometrial cancer, suggesting an involvement in endometrial cancer pathogenesis [21]. A high-resolution MS-based proteomic approach was used to identify early-stage endometrial cancer-associated proteins. This analysis using stage I endometrial cancer and postmenopausal normal endometrium tissue identified 7 out of 209 differentially expressed proteins in cancer samples regarding normal endometrium. ANXA2 and PRDX1 were considered potential biomarkers for endometrial tumorigenesis [22].

Likewise, dysregulated molecular pathways from tumor tissue samples from low-grade, early-stage endometrial cancer were reported through MS/MS proteomic analysis. A discovery and a validation cohort containing tumor and healthy samples were considered. Proteomic data identified 3112 and 9802 proteins, respectively. From the differentially expressed proteins detected in the discovery (572) and validation sets (7775), it was possible to identify a total of 854 and 5856 pathways, respectively. The authors matched the pathways identified in both cohorts, obtaining 503 cross-validated pathways. Most of these were related to cell metabolism, nucleic acid synthesis, and protein translation. This proteomic study showed changes in WNT pathways and L1CAM interaction pathways, where the CTNNB protein was upregulated in both sets. HMGB3 was the third most upregulated protein in the discovery cohort, with a consistent expression in the validation set. Additionally, a dysregulation of the SLIT-ROBO signaling pathway was found, along with the triggering necroptosis and ferroptosis pathways in these tumors [23].

Differences in the proteome of normal endometrium, atypical hyperplasia, and endometrial cancer may also help identify biomarkers of disease progression and diagnosis. However, the protein profiles of genomically unstable diploid and aneuploid endometrial cancer were similar. Also, diploid stable cancer presented a similar profile to normal endometrium. A total of 121 proteins were identified, 104 were overexpressed, and 12 were specific to endometrial cancer. These proteins were explored in atypical hyperplasia, and an increased expression of CLIC1, EIF4A1 and PRDX6, along with a reduction in ENO1, ANXA4, EMD and Ku70 expression was seen. Endometrial cancer-specific proteins were also detected in atypical hyperplasia, indicating that these proteins can be potential biomarkers of disease progression and diagnosis of endometrial cancer [24]. The comparison of the proteomic profile of different stages of the endometrioid endometrial tumor with hyperplasia and with endometrium with benign changes (BEC) tissue samples revealed significant findings. Tissue collected from stage IA endometrial cancer showed upregulation of the proteins GRP78, GSTP1, ACTG, PDIA3, and ENOA and downregulation of ALBU compared to BEC samples. Moreover, tumor tissue samples of stage IB revealed an upregulation of the proteins GSTP1, ACTB, ACTG, KRT8, ANXA1 and ENOA and a downregulation of TRFE compared to BEC tissues. Proteomic changes were also observed when comparing stage II and BEC tissue samples, where they found an upregulation of GSTP1 and PDIA3. In stage III, there was upregulation of GSTP1, ACTB, KRT8, PDIA3, TRFE and ENOA regarding controls. The comparison between hyperplasia and controls showed an upregulation of HSPB1, EF-Tu and IDH1 proteins. The proteins CALR, RPSA, ACTB, KRT8, UAP56, SOD1, PSME1, PDIA3, ANXA1, CAH1, IDHC, PPIA and PPIB presented a differential expression when all stages of endometrial cancer were compared to complex atypical hyperplasia, with downregulation of SOD1 in all endometrial cancer samples and downregulation of CAH1 and PPIB only in stage IA samples [25]. The proteomic profiling analysis in endometrial cancer, hyperplasia and healthy tissues using matrix-assisted laser desorption/ionization-time-of-flight mass spectrometry (MALDI-TOF-MS) identified 148 proteins differentially expressed between the 3 groups. Specifically, 53 proteins (28 up and 25 downregulated) were identified in malignant tissue versus controls, 26 proteins (8 up and 18 downregulated) in hyperplasia versus controls, and 32 proteins (19 up and 13 downregulated) in endometrial cancer compared to hyperplasia. In the latter comparison, DES, PPIA, and ZNF844 were downregulated, while ALDOA, ENO1, and KRT10 were upregulated. These proteins might act as potential biomarkers for an early diagnosis of endometrial cancer [26]. Identification of potential diagnostic biomarkers for endometrial cancer has revealed several novel and differentially expressed proteins. SELDI-TOF-MS analysis identified two novel differentially expressed proteins, EC1 and EC2 in endometrial malignant tissues [27]. MALDI-TOF-MS analysis identified overexpressed proteins such as CPN10 (HSP10) and S100A in endometrial cancer samples [28]. Using isobaric tags for relative and absolute quantitation (iTRAQ) in combination with multidimensional LC-MS/MS, 63 proteins were identified, with 5 differentially expressed in malignant samples: CPN10 (HSP10) and PKM were overexpressed, and SERPINA1 precursor, B-CK, and TAGLN were underexpressed, compared to controls. The cleavable isotope-coded affinity tags (cICAT) analysis identified 68 proteins, with 5 overexpressed in tumor samples: S100A11, HNRNP, MIF, PIGR precursor, and PKM. The latter was identified in both analyses. Although these methods seemed suitable for biomarker identification, validation with other conventional techniques is necessary [29]. In a subsequent study, 6 possible biomarkers identified in their previous work [29], were validated in 148 endometrial cancer tissue samples through immunohistochemistry (IHC) based on tissue microarray. CPN10 (HSP10), PKM2, and SERPINA1 were the most reliable biomarkers to distinguish endometrial tumors from normal tissues, highlighting CPN10 (HSP10) and PKM2 as good candidates for diagnostic biomarkers [30]. Further investigations into differentially expressed proteins in non-malignant and type I and II endometrial cancer samples using iTRAQ identified 1387 proteins, with 3 novel candidates suggested: WFDC2, CLU, and MUC5B [31]. Out of 17 proteins identified in subsequent research, ACTB, TUB, PK-M1/M2, 14-3-3-n and PIGR were abundant enough to be quantified. The mTRAQ-MRM approach determined the relative expression level of PIGR to be approximately 20-fold, and the PK expression levels were consistent with previous findings. All proteins were confirmed by WB [32]. Also, using iTRAQ and LC-MS/MS-based proteomics in tissue samples, 1529 proteins were identified, with 40 selected as potential biomarkers for endometrial cancer. Overexpression of CTSB, CALU, S100A6, LDHA, and HNRNPA1 in endometrial cancer tissues was validated by WB, and IHC showed an intense cytoplasmic and nuclear staining of S100A6 in tumor samples [33]. Another iTRAQ and LC-MS/MS analysis of endometrial cancer and peritumor tissue samples identified 1266 proteins. After the screening, 133 proteins were differentially expressed between tumor and peritumoral samples, with 103 upregulated and 30 downregulated. Differentially expressed proteins were allocated in KEGG pathways, identifying CCT7, HSPA8, PCBP2, LONP1, PFN1 and EEF2 as highly expressed in endometrial cancer. After validation, HSPA8 was considered the most upregulated protein, suggesting its potential as a diagnosis biomarker for early stages of endometrial cancer [34]. Using immobilized metal affinity chromatography (IMAC) and MS to analyze the phosphoproteome of endometrial cancer and control tissue samples, 31 out of 34 significantly altered proteins were increased in tumor samples, and only 3 decreased. Among these proteins, 23 have been previously identified by other authors, namely TXN, ARPC5, HBB, HSPB6, HSPB1, PSMA3, EEF1D, P4HB, CKB, PDIA6, GPI, HSPA5, ERP29, CAPZB, ANXA3, LDHB, AHCY, SNX6, WARS1, AHCY, ENO1, STIP1, and HNRNPD. Analysis of 3 type I stage 1 samples using LC-MS/MS found 552 phosphoproteins. Through 2D-DIGE analysis, 12 proteins were identified—TXN, HBB, HSPB1, PSMA3, EEF1D, P4HB, PDIA6, HSPA5, ACTG2, ENO1, H4C1, HNRNPD. Moreover, ACTG2 and H4C1 were only identified in this study. WB validation showed a significantly increased expression of HBB and HSPB1 and a decreased expression of CKB [35].

A proteogenomic analysis of an endometrial cancer cohort consisting of 138 tumors compared to 20 normal endometrial samples identified 10,135 proteins. Among these, 1292 were up, and 1488 were downregulated in tumor samples. There was an overlap of significant differences between this and exploratory cohorts [36]. PBK and KIF2C were significantly upregulated in both cohorts [37]. Type I and II endometrial cancer and normal endometrium tissue samples were analysed through LC-MS/MS to identify tumor-specific biomarkers. From 1040 spots, 33 upregulated (such as ANXA2, CAPG, and PARK7) and 9 downregulated proteins (such as CALR and UCHL1) were detected and further confirmed by WB. The overexpression of DJ-1 observed in tissues was corroborated in serum, comparing G1–G2 endometroid versus controls and serous cancer in relation to G1–G2 endometroid cancer. Similar expression levels were found between G3 endometrioid and serous cancer, indicating the potential value of DJ-1 as a detection biomarker [38]. In a recent study, 2580 proteins were identified, where 706 and 666 proteins were significantly expressed in control tissues and in malignant samples, respectively. After rigorous statistical analysis, 1848 proteins were considered. Of these, 888 proteins were common to normal and cancer endometrial tissues, 300 were only present in normal tissues and 660 exclusively from cancer samples. Among the 888 common proteins, 487 were found to be upregulated in endometrial cancer. Of the upregulated proteins in tumor samples, 67% were upregulated in both type I and II samples, with only 9% upregulated exclusively in type I and 24% exclusively in type II. Among the top 100 upregulated proteins in malignant samples, 97 were common to both types, and only 2 proteins were upregulated in type II (NCL and the PRKCSH). These data suggested that the oncogenic pathways involved in endometrial carcinogenesis are common to both types. Subsequent pathway and network analysis revealed 1 protein associated with type I (GON7) and 16 proteins expressed in type II (PAXX, BOD1L1, CAD, CCDC13, CLTB, CST3, FAM169A, GRN, MYH8, PIGT, PLCG1, PMFBP1, SARS1, SCPEP1, SLC25A4 and ZC3H4). Nine proteins were upregulated in both types of endometrial cancer samples (APP, CNPY4, GOLIM4, HEXA, JPT2, QARS1, SCARB2, SIAE and WARS1) [39]. 

Endometrioid endometrial cancer tumor samples, grades 1 and 2, and control samples were analysed with a nano-ultra-high-performance chromatography (UHPLC)-Orbitrap-MS/MS system. A total of 9042 proteins were identified, with 1445 showing differential regulation in endometrial cancer. Bioinformatics analyses showed that 10 out of the top 20 pathways were associated with human disorders and alterations in the hormonal state of endometrial cancer [40]. 

Investigating potential prognostic markers, the expression of ploidy-associated proteins in endometrial cancer cells collected from tumor samples was explored using MALDI-TOF-MS, confirmed by LC-MS/MS. Comparison between normal endometrium and diploid endometrioid carcinomas identified 19 proteins and interaction networks, with VIM, ACTB, and NFκB being the most relevant proteins. Comparison between diploid and aneuploid endometrioid carcinomas identified 20 proteins, with VIM, GRB2, and ACTB highlighted as the most important network nodes. When diploid endometrioid cancer was compared to aneuploid serous cancer, 15 proteins were identified with differential expression of ACTB, ANXA2, and HNRNPK. Eight common proteins were identified between normal endometrium with diploid endometrioid carcinomas and diploid with aneuploid endometrioid carcinomas, namely ACTB, ATP5B, ATP5E, INS, IVNS1ABP, LMNB, PLS1, and VIM. Additionally, sixteen proteins were shared between diploid and aneuploid endometrioid carcinomas and diploid endometrioid cancer and aneuploid serous cancer networks—ACTB, ACTG1, ACT, ANXA2, CAP2, EPS8L1, EPS8L2, GAS8, HIP1R, NCALD, PHACTR1, PLS1, PRS13, PRS18, SSH1, and VIL1. When comparing normal endometrium to cancer tissues, 49 proteins were identified, and NFκB, ERK1/2, and P38MAPK were considered in the main nodes [41]. The proteomic profile of three endometrial cancer tissue samples, determined using 2D-GE and MALDI-TOF-MS, showed inter-variability regarding protein identification. Each sample contained 298, 121, and 165 tumor-associated proteins. Considerable overlap was observed in functional domains between the three samples, although individual networks showed an opposite pattern, revealing the signature of each tumor. Moreover, some proteins, such as ATF2, JUN, TAF1, HNF4A, and ATF7IP, were associated with tumor aggressiveness. MST1 and PKN1 were selected for validation based on previous reports, and an increased expression of observed in non-malignant tissue compared to tumor tissue, suggesting their potential as prognostic biomarkers for endometrial cancer [42].

Using 2D-GE and a liquid chromatography–electrospray ionization tandem mass spectrometry (LC–ESI–MS/MS), proteins were identified as potential predicting biomarkers for high-risk endometrial cancer. Fresh high- and low-risk endometrial cancer and normal endometrium tissue samples were analysed, revealing twenty-two proteins. In comparing high- and low-risk samples, an increase in the PKM2, HSPA5, LMNA A/C, HRNR, and MDH2 expression and a decrease in UBE2N expression were observed. Comparing high-risk endometrial cancer versus normal endometrium, eighteen proteins showed differential expression: PKM2, HSPA5, FH, PSMC5, VIM, ALDOC, VDAC2, HNRPD, GAPDH, and EEF2 with an increased expression, and PGK1, HSPA1B, CAH1, PRDX2, C3, TF, IGHA1, and ALB with a decreased expression. PKM2 and HSPA5 were significantly increased in high-risk endometrial cancer, regarding low-risk endometrial cancer and normal endometrium, suggesting they are potentially predicting risk biomarkers for endometrial cancer [43]. A multi-omic characterization of endometrioid and serous endometrial cancer and normal tissues was conducted, classifying tumor tissues into four genomic subtypes: POLE, MSI, CNV-low, or CNV-high. Significant differences in the protein and post-translational modification levels between these genomic subtypes were identified. The functional analysis revealed increased expression of proteins involved in cell transport and metabolism, along with downregulation of cell cycle proteins and phosphorylation in the CNV-low subtype. An increase in phosphorylated proteins involved in ATM signaling, and suppression in mismatch repair proteins was observed in the POLE, MSI and CNV-high subtypes. Serous samples presented the highest upregulation in ribose biogenesis pathways, associated with poor cancer prognosis. MLH1 and EPM2AIP1 were downregulated in MSI samples at both protein and mRNA levels, while PMS1 and PMS2, showed decreased protein levels. An upregulation of RPL22L1 in MSI tumors was noted at both mRNA and protein levels, with a mutation in its paralog gene *RPL22* present in most of the MSI tumor samples [36]. A proteomic approach identified potential markers for endometrial cancer in patients previously treated with tamoxifen for breast cancer. Tumor samples from patients who developed endometrial cancer during or after adjuvant tamoxifen treatment and those from patients with primary endometrial carcinomas without tamoxifen treatment were used in this analysis. A total of 904 proteins were identified, revealing a clear proteomic profile distinguishing tumors from normal tissue samples. Comparing tumor samples with normal tissues, 431 upregulated and 115 downregulated proteins were identified. CAPS, PRTN3, HMGA2, PKM, AZU, ANXA2, CTSB, SFN, S100A8, LTF, CTSD, and STMN1 were most abundant in tumor tissues while CNN1, CDH13, CALD1, DES, and TAGLN presented lower abundance in tumor tissues compared to normal tissues. A total of 6 proteins were differentially increased in tamoxifen-treated samples, including HMGA1-2 and STMN1, while 22 proteins, including AZU1, PRTN3, TAGLN, CALM, CAPS, CTSG, and CDH13, were more abundant in the tamoxifen untreated samples. Invasive and non-invasive tumors were also compared, with 14 up and 32 downregulated proteins in invasive tumors. PRTN3, AZU1, CTSG, CAPS, S100A8, and ANXA2 were highlighted among the upregulated, and STMN1 was among the downregulated. Type II and I tumors revealed 50 and 38 proteins with increased and decreased expression, respectively, with cytoskeletal proteins CNN1, TAGLN, DES, CALD1, and CDH13 being less abundant in type II tumors. High levels of STMN1 were suggested to be related to poor survival in endometrial cancer patients [44].

Racial disparities are a reality in many types of cancer, which can influence their progression and prognosis. A proteomic profile analysis of endometrial cancers from Black, White, American Indian, and Asian groups with the same age, BMI, and histology identified 1611 proteins across all samples. Among these, 58 proteins showed significant expression differences among the races. EIF4G2, F13A1, GFM1, NPEPL1, SARS2, SNTB1, UBR4, USP47, and WDR5 were distinct across all races. ASS1 was significantly higher in American Indian patients compared to White patients. PFAS was elevated in Black and White patients. Another protein involved in metabolism, CKB, had higher expression in Asian patients compared to White patients. HK2 was elevated in Black and American Indian groups, with the lowest expression in the White group. Two kinases, MAPKAPK3 and OXSR1, and a phosphatase, PTPN6, were also present at different levels in the races. MAPKAPK3 was present at higher levels in Black patients compared to White patients. OXSR1 was highly expressed in Black patients, with the lowest expression in Asian patients. PTPN6 had the highest levels in Black patients and the lowest in Asian patients. EIF4A2 was elevated in the Black group compared to the White group. The serine protease inhibitor SERPINA1 was highly expressed in Asian and American Indian patients, with the lowest expression in Black patients. These findings may shed light on racial disparities in endometrial cancer and contribute to more tailored treatments based on race, potentially improving treatment responses [45]. Investigating predictive biomarkers for metastasis using a proteomics-based approach (LC-MS/MS) has identified key differences in protein expression between primary and metastatic endometrial cancer. In primary endometrial cancer, 42 proteins were identified, while brain metastasis samples revealed 53 proteins, with 27 common to both. Among these, TPI1 expression was higher in metastatic tumors, while TAGLN2 was more abundant in primary tumors. The metastatic tumors also expressed higher levels of ENO1, ATP5A, and TUBB [46]. Additionally, in another analysis of 60 selected proteins in 10 endometrial cancer tissue samples with or without lymph node metastasis (LNM), 23 proteins were identified, with ANXA2 showing higher expression in samples with LNM. In contrast, ERBB2, EGFR, and ACTN4 had lower expression in these samples. ANXA1 was also recognized for its role in the dissemination process. Thus, the identified biomarkers could be used in LNM prediction models for endometrial cancer [47]. The kinase proteome profile of endometrioid and serous endometrial tumors, compared to normal endometrial tissues identified 347 kinases, where SRPK1 overexpression in tumor samples was associated with a worse prognosis. This finding suggested that targeted therapeutic strategies focusing on SRPK1 could be a promising anticancer approach [48].

Samples from endometrial cancer patients stratified as responders and non-responders to metformin treatment were examined to explore a therapeutic predictive marker. Out of 1289 identified proteins, 79 were significantly altered between responders and non-responders. Pathway analysis revealed alterations in the PRKAA2, also known as the AMPK signaling pathway, along with modifications in pathways related to cellular signaling activation, regulation of cell proliferation, and inhibition of cell death and apoptosis, in tissues from metformin responders. Significant protein alterations were also observed when comparing pre-treatment tissues from responders to non-responders, which correlated with changes in post-treatment tissues from responders compared to pre-treatment tissues. Eleven proteins (ACTA2, TPR, MAP4, HBG2, PSMD11, SLC2A11, SLC2A1, SRRM2, U2AF1, TMSB4X, and DVL-2) were identified as altered. JPT1 was further validated as a predictive and pharmacodynamic biomarker due to its significant fold change in pre-treatment biopsies from responders versus non-responders and its decreased abundance in post-treatment tissues in metformin responders [49]. In fact, HSPA8 has already been considered a therapeutic biomarker for early-stage endometrial cancer [34].

Tumor heterogeneity poses significant challenges in cancer research. Protein composition analysis of different regions within endometrial cancer tissues from 63 samples of 20 patients revealed notable heterogeneity in 3 patients, suggesting that differentially expressed proteins could serve as promising biomarkers to explain intra-tumor variability. Analyzing the proteomic profile of samples from pre- and postmenopausal patients, 1985 proteins were identified, with 5.8% showing higher expression in postmenopausal patients. Notable upregulated proteins in postmenopausal women included EWSR1, TUBA1A, TIGAR, SEC11A, and CENPV, while TMSB4X, COL1A2, S100A16, NEBL, and OGN were downregulated. The Human Protein Atlas database also showed an upregulation of EWSR1, TUBA1A, TIGAR, CENPV, COL1A2, S100A16, and NEBL in endometrial cancer compared to normal tissues. Proteomic changes associated with myometrial invasion, a marker of cancer aggressiveness, identified 79 proteins unique to highly invasive tumors, and 22 unique to less invasive tumors. Fifteen proteins were significantly overexpressed in highly invasive tumors, with EWSR1, TIGAR, SLC9A3R1, DNAJB11, and RBBP4 as top 5 candidates. In deep myometrial invasion (>10%), 48 proteins, including MYH11, NEBL, COL1A2, OGN, and GNLY, were downregulated. Comparing grades 1 and 2, 1860 common proteins were identified, with eight significantly upregulated in grade 2—EWSR1, MZB1, Mx1, NANS, TMED9, TPPP3, HNRNPF, and NOLC1. Additionally, 26 proteins were downregulated in grade 2, highlighting HBA1, COL1A2, SLC4A1, COL5A1, and FGA. In high-grade serous tumors compared to grades 1 and 2, 1632 differentially expressed proteins were identified. A total of 30 proteins were significantly upregulated in high-grade serous tumors, including SNRPC, UBE2V2, COL1A1, BCAM, and PTMA, while DEFA1, S100A8, LTF, CAMP, and AZU1, were among the 18 downregulated. Comparing high-grade serous and grade 2 tumors, 288 proteins were unique to grade 2. Six proteins, namely SNRPC, COL1A1, COL1A2, SEC63, LDHB, and ABHD14B, were significantly more expressed in high-grade serous tumors, while LTF, GSTP1, SARS1, ATP1B1, IARS1, PNP, and SFN were downregulated. High EWSR1 protein expression is particularly notable in invasive tumors, especially in postmenopausal patients, suggesting its potential as a biomarker for aggressive endometrial tumors in older women [50].

In uterine aspirates from endometrial cancer patients and healthy controls, 52 potential biomarkers were explored using LC-MS/MS. The analysis revealed an increased expression of 26 proteins in cancer samples. ROC analysis identified ten proteins with high performance as diagnostic biomarkers for endometrial cancer: MPO, CADH1, SPIT1, ENOA, MMP9, LDHA, CASP3, PKM, PRDX1, and OSTP isoform A. Among these, MPO, CADH1, SPIT1, and OSTP isoform A exhibited the greatest performance [51]. The same panel of 52 biomarkers was evaluated in the fluid fraction of uterine aspirates using LC-MS detection with the parallel reaction monitoring technique (LC-PRM). This study identified 28 differentially expressed proteins in endometrial cancer samples, with LDHA, PKM, MMP9, NAMPT, and SPIT1 showing the best performance in distinguishing endometrial cancer from normal tissues. These proteins, along with MPO, were good markers for early endometrial cancer diagnosis. Additionally, NAMPT, ENOA, CATD, and GSTP1 were differentially found between endometrial cancer and control samples, enabling the distinction of hyperplasia cases. For diagnostic biomarker panels, MMP9 and PKM were reliable for discriminating endometrial cancer, while the combination of CTNB1, XPO2, and CAPG revealed higher performance in distinguishing endometrioid from serous endometrial cancer types [52]. Uterine aspirates analysed with MALDI-TOF and LTQ-Orbitrap XL revealed 25 proteins, with 15 showing the best performance (sensitivity and specificity 100%). ABRACL, PGAM2, FGB, and ANXA3 were identified as endometrial cancer-specific proteins, and their expression was validated through WB. ABRACL and PGAM2 were indicated as the most promising biomarkers for endometrial cancer diagnosis [53].

**Table 1:** Studies using endometrial tissue, uterine aspirates and sentinel lymph node samples.

Ref.	Sample Number	Age	Normal Samples	Pathological Samples	Methodology	Upregulated Proteins	Downregulated Proteins	Validation
[20]	44	ND	Normal endometrium (containing atrophic, proliferative, secretory, and menstrual, benign endometrial polyp and disordered proliferative *n =* 23)	Endometrial cancer (endometrioid, mucinous, and serous adenocarcinomas, and malignant mixed Mullerian tumors *n =* 21)	SELDI-TOF-MS	HSP10	NA	WB and IHC
[28]	16	ND	Normal endometrium (secretory *n =* 4, proliferative *n =* 4)	Endometrial cancer (endometrioid *n =* 8)	MALDI-TOF-MS	HSP10, S100A	NA	ND
[27]	39	36–63 years	Normal endometrium (*n =* 20)	Endometrial cancer (Grade 1–3, Stage I–III; *n =* 20)	SELDI-TOF-MS	EC1	EC2	ND
[29]	8	ND	Normal endometrium (secretory *n =* 1, proliferative *n =* 2)	Endometrial cancer (*n =* 5)	iTRAQ, cICAT, LC-MS/MS	PKM1, PKM2	NA	ND
[31]	39	ND	Normal endometrium (secretory *n =* 10, proliferative *n =* 10)	Endometrial cancer (type I *n =* 10, type II *n =* 9)	iTRAQ and MS/MS	WFDC2, CLU, MUC5B	NA	Dot-blot and IHC
[22]	91	ND	ND	Endometrial cancer (endometrioid *n =* 79, serous *n =* 12; Grade 1–3, Stage IA, IB, IC)	LC-MS/MS	ANXA1, ANXA2, PRDX3, RDX4, PRDX5, PRDX6, COX2	NA	TMA and WB
[33]	20	ND	Normal endometrium (proliferative *n =* 10)	Endometrial cancer (Type I *n =* 10)	SCX separation and RP LC-MS/MS and iTRAQ	CTSB, CALU, CACYBP, LDHA, HNRNPA1	NA	WB and IHC
[41]	18	36–92 years	Normal endometrium (*n =* 4)	Endometrial hyperplasia (*n =* 14)	2D-DIGE and MALDI-TOF/TOF	NFκB, ERK1/2, P38MAPK	NA	LC-MS/MS
[24]	40	ND	Normal endometrium (*n =* 8), Squamous epithelium (*n =* 4)	Endometrial cancer (endometrioid *n =* 15, serous *n =* 13)	2D-DIGE and MALDI-TOF-MS	EIF4A1, CLIC1, PRDX6	CLIC4, ENO1, ANXA4, EMD	IHC
[46]	1	ND	NA	EC FIGO stage IB (*n =* 1), brain metastasis (*n =* 1)	LC-MS/MS	TPI1, TPI-1, TAGLN2	NA	WB and IHC
[42]	3	ND	NA	Endometrial cancer (endometrioid stage IA type I *n =* 3)	2D-GE and MALDI- TOF-MS	ATF2, JUN, TAF1, HNF4A, ATF7IP	NA	IHC
[43]	15	50–77 years	Normal endometrium (*n =* 5)	Endometrial cancer (high-risk *n =* 5, low-risk *n =* 5)	2D-GE and LC-ESI- MS/MS	PKM2, HSPA5	NA	IHC
[34]	10	ND	Adjacent normal tissue (*n =* 10)	Endometrial cancer (stage I *n =* 10)	iTRAQ and LC- MS/MS	HSPA8	NA	WB
[47]	10	ND	NA	Endometrial cancer (*n =* 10)	LC-ESI-MS/MS and MALDI-MSI	ANXA2, ERBB2, EGFR	ACTN4, ANXA1	TMA and IHC
[49]	20	ND	NA	Endometrial cancer (obese *n =* 20)	LC-MS/MS	JPT1	NA	IHC
[25]	30	ND	Benign endometrial (*n =* 7)	Endometrial cancer (Complex atypical endometrial hyperplasia, *n =* 2; endometrioid type adenocarcinoma (stage IA *n =* 5, stage IB *n =* 5, Stage II *n =* 3, stage III *n =* 5))	2D-DIGE and MALDI-TOF/TOF	CALR, RPSA, ACTB, KRT8, UAP56 (DDX39R), PSME1, PDIA3, ANXA1, IDH1, PPIA	SOD1, CAH1, PPIB	NA
[36]	95	ND	NA	Endometrial cancer (endometrioid *n =* 83 and serous *n =* 12)	LC-MS/MS	NA	MLH1, EPM2AIP1	NA
[48]	36	ND	Normal endometrium (*n =* 16)	Endometrial cancer (endometrioid *n =* 17, serous *n =* 3)	MIB-MS and nano-LC-MS/MS	SRPK1	NA	IHC
[44]	45	ND	Normal endometrium (*n =* 11)	Endometrial cancer (grade I *n =* 20, grade II *n =* 8, grade III *n =* 6)	LC-MS/MS	CAPS, PRTN3, HMGA2, PKM, AZU1, ANXA2, CTSB, SFN, S100A8, LTF, CTSD, STMN1	CNN1, CDH13, CALD1, DES, TAGLN	NA
[21]	6	ND	Normal endometrium (*n =* 3)	Endometrial cancer (clear cell or type 2 carcinoma *n =* 2, and carcinosarcoma *n =* 1)	LC-MS/MS	IFIT3, PARP9, SLC34A2, CYB5R1, PTPN1	DPT, SLP1	RT-qPCR and WB
[23]	32	66.5–78 years	Normal endometrium (*n =* 16)	Endometrial cancer (*n =* 16)	TMT-Labelling and LC-MS/MS	Wnt pathway, L1CAM, β-catenin, HMGB3, SLIT/ROBO pathway	NA	Cohort and RT-qPCR and TMA and IHC and Immunofluorescence
[40]	87	ND	Normal endometrium (hysteromyoma, cyst, endometrial polyps and cervix diseases *n =* 43)	Endometrial cancer (type I grade 1–2 *n =* 44)	LC-MS/MS	NAAG2, GCPII, NAAG, NAA, GSSG, GSR, DBH, BCAT1, PK, AK2, AMPD3, IMP	GSSG, CDA	NA
[45]	46	61.2 years	NA	Endometrial cancer (African American *n =* 12, Whites *n =* 12, Native American *n =* 12 and Asian *n =* 10)	TMT-Labelling and LC-MS/MS	PFAS, EIF4A2, MAPK3, CKB, HK2, PTPN2	ASS1, OXSR1	NA
[26]	36	46–75 years, age-matched	Normal endometrium (adenomyosis, fibroids, hormone imbalance *n =* 12)	Endometrial cancer (*n =* 12) and hyperplasia (*n =* 12)	2D-DIGE and MALDI-TOF/TOF	ALDOA, ENO2, KRT8	DES, PPIA, ZNF844	LC-MS/MS and MRM Transitions
[50]	63	43–84 years	NA	Endometrial cancer (endometrioid *n =* 18, serous *n =* 2)	SWATH-MS and LC MS/MS	EWSR1	NA	NA
[37]	158	64 years	Normal endometrium (*n =* 20)	Endometrial cancer (endometrioid *n =* 119, serous *n =* 13, clear cell *n =* 3)	LC-MS/MS	PBK, KIF2C	NA	IHC
[39]	8	55–88 years	Normal atrophic endometrium (*n =* 4)	Endometrial cancer (endometrioid *n =* 2, serous *n =* 2)	LC-MS/MS	APP, CNYP4, GOLIM4, HEX4, JPT2, QARS1, SCARB2, SIAE, WARS1	NA	NA
[35]	26	59–74 years	Normal endometrium (*n =* 13)	Endometrial cancer (*n =* 13)	2D-DIGE and LC-MS/MS	HBB, HPSB1 LDHB	CKB	WB
[51]	42	>50 years	Normal endometrium (*n =* 20)	Endometrial cancer (*n =* 22)	LC-PRM	NA	NA	NA
[52]	116	ND	Normal endometrium (*n =* 47)	Endometrial cancer (endometrioid *n =* 49 and serous *n =* 20)	LC-PRM	LDHA, PKM1/M2, MMP9, NAMPT SPIT1	NA	ELISA
[53]	16	ND	Normal endometrium (*n =* 6)	Endometrial cancer (*n =* 10)	2D-GE and MALDI-TOF/TOF	FGB, ENO1, ANXA3, PRDX2, GAPDH, PSMB6, GSS, ASRGL1, PGK1, CORO1A, PSME1, PDIA3, IDH1, LDHB	NA	WB
[38]	5	ND	Normal endometrium (*n =* 5)	Endometrial cancer (endometrioid Grade 1–2, *n =* 5)	2D-DIGE and LC-MS/MS	ANXA2, CAPG, PARK7	CALR, UCHL1	IHC
[54]	36	N—44–70 years, EC—59–74 years	Endometrium (nodes *n* = 3, tissue *n* = 6)	Endometrial cancer (nodes *n =* 16, tissue *n =* 16)	LC-MS/MS	PRSS3, ASS1, PTX3, ANXA1	NA	IHC
[32]	24	NA	Normal endometrium (*n =* 14)	Endometrial cancer (*n =* 10)	SCX, nano-LC-MS, MRM transition	ACT, TUB, PK-M1/M2, 14-3-3-n, PIGR	NA	NA

Abbreviations: ND, Not Defined; NA, Not Applicable; TMA, Tissue Microarray; ELISA, enzyme-linked immunosorbent assay.

### 3.2. Sentinel Lymph Node Tissue

The only analysis of SLN and corresponding tissue identified 1005 proteins among 36 samples of women with and without endometrial cancer (Table 1). Of these SLN samples, 3 proteins were specific to normal, 21 proteins from grade I, 5 proteins from grade II, and 33 proteins from grade III. Across all groups, 336 proteins were differentially expressed. Comparing all SLN grades to normal SLN, 91 proteins were differentially expressed, with 44 proteins overexpressed in cancer SLN samples and 47 proteins in normal SLN. Grade I overexpressed proteins involved in innate immune response and energy pathways, such as DCD, S100-A8, S100-A9, RNASE3, LYZC, LTF, ELANE, CTSG and LCN2. Grade II overexpressed 37 proteins including IDH1, SHMT2, OGDH, ADP/ATP, UGDH, NANS, HSPA5, HYOU1, HSP90A1 and PDIA3. Grade III overexpressed 32 proteins including ICAM-3, CTNNB1, STMN family and TP53BP1. Normal SLN overexpressed 26 proteins involved in oxygen carrier activity, oxygen binding and myosin binding. In the analysis of tissue, 913 proteins were identified. Of these, 3 proteins were specific to healthy endometrium, 24 to grade I tissue, 14 to grade II and 19 to grade III. Across all groups, 384 proteins showed significant expression differences. Grade I tissue overexpressed 30 proteins involved in immune response, such as IFI16, LYZC, MPO and LTF. Grade II tissue overexpressed 42 proteins involved in cytoskeleton protein binding, ribosome, and actin-binding, including TPM4, TPM2, TPM1, MYH10, and MYH11. Grade III tissue overexpressed proteins including CDH13, CTNNB1, TNN, VTN, EMILIN-1, COL2A1, APOE and HSPG2. Lastly, comparing SLN and endometrial cancer tissue grades, a positive correlation was found between grade I of both tissues with the proteins PTMA, ACTL6A, SHMT2, RBM25, and RBM4. SUB1 and ETHE1 were expressed in grades II and III, DCD in grades I and II, and YBX2, NOTUM and RANDBP1 in grade III. NUP210 was specific to grade III, while PADI4, MUC5B, GOLM1, MNDA, CHI3L1, PTX3, SP100, MMP8, AZU1 and SLC9A3R2 were specific to grade I. Markers common to both SLN and tissue were grade dependent, with PRSS3 in grade III, ASS1 in grade II and PTX and ANXA1 in grade I. ALDH2 was present in grades II and III. PTMA, ACTL6A, SHMT2, RBM25, and RBM4 were detected in grades I, II and tissue grade III; SUB1, and ETHE1 in grades II and III; DCD in grades I and II; YBX2, NOTUM, RANDBP1 in grade III; NUP210 in grade II; PADI4, MUC5B, GOLM1, MNDA, CHI3L1, PTX3, SP100, MMP8, AZU1, SLC9A3R2 were specific to grade I [54].

### 3.3. Serum and Plasma

Protein fragments and peptides generated in endometrial tissue can be subsequently released into the bloodstream, providing valuable insights about potential biomarkers. Ten studies have focused on investigating possible serum biomarkers for diagnosing endometrial cancer, which are detailed in Table 2.

An analysis of serum high-abundance proteins among patients with endometrial stage IA or IB compared to negative control women revealed significant alterations. The analysis was confined to 12 clusters of protein spots with nominal mass (Mr) ≥ 30,000—SERPINA1, ABCG, ACT, COG4, ATR, CLU family, HBB, KNG1, LRG1, and AZGP1. In endometrial cancer patients, the expression of ABCG, ATR, CLU, and LRG1 was upregulated, while the expression of SERPINA1 and KNG1 was downregulated. These findings were validated using competitive ELISA and lectin methods [55].

Proteomic analysis on serum samples from untreated endometrial cancer patients and healthy controls after depletion of highly abundant proteins identified 49 differentially expressed peaks. Eleven of these peaks corresponded to protein sequences in the NCBInr database, specifically APOA4, ITIH4, C3, C4A, and C4B. APOA4 was downregulated in endometrial cancer patients, while C4A and C3 were upregulated. These findings were validated through immunoblotting using serum samples from five endometrial cancer patients and five healthy controls [56]. The proteomic analysis compared patients with distinct stages of endometrial disease, including simple endometrial hyperplasia (SEH, *n =* 6), complex hyperplasia (CEH, *n =* 4), atypical hyperplasia (AEH, *n =* 4) and early-stage (ES, *n =* 6). Twelve proteins showed significantly altered expression in at least one disease group. Among these, seven proteins were differentially expressed in AEH: ORM1, HP, SERPINC1, SERPINA3, APOA4, ITIH4, and HRG. Two proteins, SAA and SAA2, showed significant elevation in ES as compared with the normal control. Three additional proteins, APOC2, APOE (both upregulated) and APOA4 (downregulated), showed consistently altered expression with high confidence levels in all four disease groups. HRG was also downregulated in all four disease groups, while HP was upregulated in AEH and EC but downregulated in CEH and SEH. IGFBP-4 was significantly upregulated in SEH and mildly in CEH and EC. These findings provide insights into protein expression variations across different stages of endometrial disease, though the limited sample size [57]. Serum proteins from 15 endometrial cancer patients before treatment were compared with 15 controls, identifying 16 proteins with diagnostic potential. Six of them were upregulated in endometrial cancer patients (CLU, SERPINC1, ITIH4, C1RL, APOC3 and DSC1), while ten were downregulated (APCS, C9, APOA1, ALB, APOA4, CFHR1, ITIH2, and ACTB). Validation confirmed the upregulation of CLU, ITIH4, SERPINC1 and C1RL. A predictive model using 3 of the proteins (C1R was excluded) achieved a sensitivity of 100%, specificity of 86% and AUC 0,93 for predicting endometrial cancer [58]. Proteomic analysis of sera from 10 endometrial cancer patients and 10 healthy controls revealed significantly different expression of 24 proteins. A total of 7 proteins were upregulated (APOC3, APOC2, SERPINC1, C1R, SERPINA1, A2M, CLU) while 17 were downregulated (APOA1, APCS, APOE, CD5L, CFHR1, VTN, C9, C8A, ALB, C4BPA, IGHM, ITIH2, FLG2, SBSN, APOA4, CPS1), respectively. Downregulation of SBSN in the serum of patients was further validated by WB and in silico analysis of the TCGA database. The study does not clarify whether the population and samples used for the proteomic analysis overlap with those from the team’s previous investigation [58,59].

An analysis of serum from patients with endometrial cancer across all stages and different histological types identified 9157 protein peaks. Among these, four biomarkers were selected to differentiate endometrial cancer patients from healthy women. Two biomarkers (APOA1 and its modified form) were downregulated, while two (APOC1 and its modified form) were upregulated in endometrial cancer patients; these differences were also significant for stage I patients. Dual marker analysis showed a sensitivity of 78% and a specificity of 90% for identifying endometrial cancer patients. Validated with a blind test set resulted in a sensitivity of 82% and a specificity of 86% [60]. Serum samples from early endometrial cancer patients and patients with benign pathology were compared before and after surgery, identifying 17 proteins uniquely expressed in endometrial cancer patients’ sera before surgical treatment. Among these, FAM83D showed the greatest potential as a biomarker, validated using WB analysis on cell lysate preparations and tumor tissue specimens from endometrial cancer patients. However, the small sample size limits the study’s findings [61]. Using an LFQ proteomics approach, the exosome proteome from the albumin-depleted serum of early endometrial cancer patients and non-tumor controls was investigated. A total of 33 proteins exhibited significantly different expression levels in cancer patients: 31 proteins displayed increased levels in tumor samples (CAH1, HBD, HBB, LPA, PF4V1, SAA4, APOA1, APOE, HBA1, C1QC, C4BPB, APOC2, VTN, FGA, ORM2, APOB, PROS1, SERPINA3, CLU, APOD, F2, SERPINF2, GPX3, AZGP1, SERPING1, PON1, A2MG, LGALS3BP, AFM, APOA4, and PZP). Only two proteins, IGLV3–19 and IGKV3–20, showed lower levels. WB analysis of serum and tumor tissue specimens confirmed the upregulation of eight proteins: APOA1, HBB, CAH1, HBD, LPA, SAA4, PF4V1, and APOE. A predictive model incorporating these 8 proteins, achieved a sensitivity of 100% and specificity of 86.11% in discriminating stage 1 patients from controls, though the model’s performance was less satisfactory in identifying endometrial cancer patients at more advanced stages [62]. A xenograft model was utilized to explore potential tumor biomarkers for endometrial cancer patients. Healthy female nude mice were subcutaneously inoculated with the human endometrial carcinoma cell line HEC-1-B. Proteomic analysis of serum samples identified over 224 proteins, with 175 (78.1%) originating from mice, and 45 (20.1%) unclassified as either human or mouse origin. FRAS1 was identified as a uniquely human-origin protein. WB then confirmed its expression profile of FRAS1 in serum samples from xenograft mice and endometrial cancer patients, with no expression in healthy controls [63]. Histidine, tryptophan, valine, phenylalanine, asparagine, serine, leucine, and methionine were significantly lower in endometrial cancer samples; while ornithine, isoleucine and proline were significantly higher. A model for detecting endometrial cancer based on histidine, isoleucine, valine, and proline was developed, the plasma amino acids profile (PAAP), showing better performance than serum CA125. PAAP achieved a sensitivity of 60% to detect endometrial cancer, meanwhile, CA125 was 22.5%. PAAP demonstrated an AUC of 0.91 for detecting stage I early endometrial cancer and 0.98 for advanced stages, compared to 0.79 and 0.83 for CA125, respectively. A validation cohort confirmed PAAP’s effectiveness, detecting endometrial cancer in 11 out of 17 advanced-stage samples and 13 out of 23 early-stage samples, whereas CA125 detected only 5 out of 17 advanced-stage samples and 4 out of 23 early-stage samples. Even though they were able to correlate PAAP expression with disease stage, there was no correlation established between PAAP and age or body mass index [64].

### 3.4. Cervicovaginal Fluid

Two papers describe the analysis of cervicovaginal fluid (CVF) through proteomic techniques to profile endometrial cancer, and their details are detailed in Table 3.

Analysis of CVF samples identified 2425 proteins with regulatory, cytoskeletal, and immune functions. There was a 20% commonality between the CVF and cell lines samples and approximately 650 proteins were exclusive of CVF being extracellular, immune-related and with acute inflammatory properties. A total of 269 proteins were unique to the supernatant of endometrial cancer samples, 92 were unique to the pellet, and 37 were common in both pellet and supernatant. In atypical hyperplasia samples, 31 proteins were unique to the supernatant, and 73 proteins were unique to the pellet. A small cohort of 15 women was used for validation thus identifying 680 proteins, with over 200 being unique to this analysis. Among these, several proteins associated with endometrial cancer were found, such as HSP10, HSP60, HSP71, HSP75, S100A8/9, FABP5P3, PK, PGAM1, ENO1, SERPINA1, ADIPOQ, APO, SAA, MMP-9, MUC-1, MUC-16, HE-4, A1BG, ALB, TFR, IgGs, DEFA family, CRISP-3, DCD, CAMP and CRP [65]. Comparative analysis of CVF samples from normal endometrium with endometrial cancer, identified 1600 to 1800 proteins in endo-, exocervical, and uterine fluid. From a list of 506 proteins described in the literature as potential endometrial cancer diagnostic biomarkers, 171 proteins were identified in this study. A total of 14 of the 20 most validated biomarkers in tissue samples were detected in all samples, including HE4 and CA125, the 2 most studied diagnostic biomarkers for endometrial cancer. Among 52 proteins previously identified by the research team, 8 proteins (MMP9, KPYM, LDHA, CADH1, NAMPT, MPO, ENOA and CAPG) achieved the highest accuracy in diagnosing endometrial cancer in CVF [66].

### 3.5. Urine

The urine proteomic profile for endometrial cancer was described in three papers, detailed in Table 4.

Urine samples from newly diagnosed endometrial cancer and healthy women that were resolved in 2-DE maps originating seven clusters were identified as KNG1, MPG, AZGP1, CD59, AMBP, IgG3C, and IgKC [67]. In another study, urine samples of endometrial cancer and healthy patients identified 181 proteins with different regulations, where 76 were statistically significant changes. Regarding these proteins, HSPG2, VTN and CDH1 may play a significant role in being potential biomarkers of endometrial cancer [68]. Starting from 798 proteins quantified from a cohort of 104 women, where CSTA, CDC5L, FLG, TPD52L2 and HSP90B1 were among the top 10 most significant. To effectively differentiate between endometrial cancer and normal endometrium, a 4-biomarker panel with CSTA, S100A7, MMP9 and SERPINA10 and a 10-biomarker panel adding RTN4, LAMP2, WDR1, KRT13, ALDH2 and ILF were created showing elevated specificity. Due to the proximity of the uterus, some uterine-derived biomarkers can contaminate the urine samples. Despite this, the best performing diagnostic model was a 10-marker panel combining SPRR1B, CRNN, CALML3, TXN, FABP5, C1RL, MMP9, ECML1, S100A7 and CFI, which predicted endometrial cancer with AUC of 0.92 [69].

### 3.6. Endometrial Cancer Cell Lines

In vitro studies describing the proteome in endometrial cancer were described in six papers, detailed in Table 5.

A 2D model of endometrial cancer cell lines, analysed with 2D LC-MS/MS, revealed 198 proteins in KLE cells and 87 in HEC-1 cells. Subsequent validation was performed in 148 tissue samples by LC-MS/MS, presenting HSPE1, PK-M1/M2, SERPINA1, S100-A11, and MIF proteins as potential biomarkers. HSPE1, SERPINA1 and PKM2 constitute the panel of proteins that satisfactorily differentiate endometrial cancer and normal endometrium. Additionally, some proteins linked with other cancers were also found in this study, such as KLK10, MSLN, IGFBP2/3/4/6/7/10, GDF15, TGF-b, CLU family, WFDC2, PLK1, RTN4, OPN, SPARC, CALU family, CFL family, AGRN family, TIM1/2, CSTB and CST3 [70]. Using iTRAQ and LC-MS/MS, analysis of 3 normal endometrial cell lines and 7 endometrial cancer cell lines 272 proteins were identified, from which 139 proteins were in the plasma membrane. In 4 of 7 endometrial cell lines, there were 11 proteins with increased expression compared to the normal endometrial cell line. BST2 was the protein that showed the most significant differences in expression between normal and endometrial cancer cells. IHC was used to confirm the expression of BST2 in a cohort of 177 patients as a potential biomarker for endometrial cancer [71]. In the analysis of HEC-1A and Ishikawa 2D and 3D cultures, 5735 proteins were quantifiable. HEC-1A had 186 proteins upregulated and 93 downregulated, and Ishikawa had 154 upregulated and 81 downregulated. Both cell lines had 167 proteins in common, of which 43 were upregulated, and 124 were downregulated. The most affected pathways were associated with the HIF-1 signaling pathway, ECM-receptor interaction, PI3K-Akt signaling and glycolysis/gluconeogenesis. The potential proteins found in LC-MS/MS were validated with WB, which corroborated the finding in the proteomic approach and the elevated expression of PFKFB3, GPRCA5 and HK2 [72]. Whole-cell protein lysates established from primary epithelial cancer cell lines from endometrial adenocarcinoma and a 3D culture were analysed using 2D-DIGE, resulting in 78 proteins differently expressed between 2D and 3D cultures. The comparison of proteins associated with 2D and 3D may suggest which proteins are related to the biological characteristics of the cells. Of 22 proteins differently expressed, 8 were upregulated (i.e., PRDX1, VDAC1, PHB family, ANXA4) and 14 downregulated (i.e., TUBB, VIM, PRDX6) in the 3D vs. 2D cultures [73].

The increased expression of the PHB family in 3D culture supports the reduced cellular proliferation observed, the differences in VDAC1 and ANXA4 expression support that 3D culture is more apoptosis prone, and lastly, reduced expression of TUBB, VIM and TKT family was also found [73]. Human endometrial stromal cells (HESC) and the Ishikawa cell line were used in a 3D co-culture model. The cell lines were used in a 3D model to later be compared to the proteins found in tissue samples. Between 3D co-culture, 3D Ishikawa cells and normal endometrium tissue, there were identified 1618 proteins, where 1185 proteins were from 3D co-culture, 500 proteins were common in all samples, and 91 proteins were exclusive to normal tissue and 3D co-culture. From the 91 proteins identified, IPA analysis revealed 10 out of 81 relevant pathways, like RhoA signaling, ITG signaling, EPH signaling, PDGF signaling, VEGF signaling, INSR signaling, EGF signaling, ErbB2/3/4 signaling, and BMP signaling. Next, to validate the involvement of the proteins in these 10 canonical pathways, 3D co-culture and endometrial samples, thus revealing 8 proteins. These 8 proteins revealed still different expression patterns for ARPC2, PPP1R12A, MAPK1, GRB2, EIF2AK2, and EIF2S2 [74]. Proteomic analysis of Ishikawa and HEC-1A identified 6003 proteins in both cell lines, with 105 proteins absent in the PAN human library. Cervical-vaginal fluid samples were also examined with 1186 common proteins [65].

### 3.7. Analysis of Potential Biomarkers

**Figure 3:**
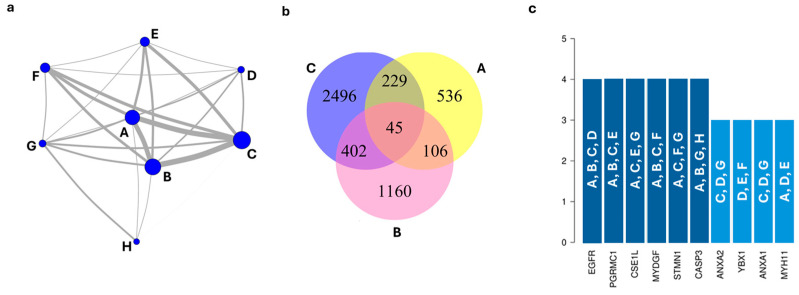
Consistency of potential biomarkers across studies. (**a**) Network of potential biomarker overlap proposed in different studies. The edges correlate with the number of shared potential biomarkers from the connected studies. Node sizes indicate the number of potential biomarkers proposed by the specific study. (**b**) Venn diagram displaying the number of proteins shared by the three studies most strongly connected in the network. (**c**) Bar plot indicated the most frequently reported potential biomarkers, identified in each study as a regulated protein (A [21], B [40], C [36], D [47], E [49], F [39], G [66], and H [52]).

Due to the quantity and diversity of proteins identified in the various studies using endometrial tissue, an analysis of eight studies (A [21], B [40], C [36], D [47], E [49], F [39], G [66], and H [52]) was carried out to evaluate the consistency of the potential biomarkers proposed (Figure 3). The network analysis showed that three studies, A, B and C were the most strongly connected, sharing a considerable number of proteins, corresponding to those with the highest number of potential biomarkers suggested (Figure 3a).

The total number of identified proteins in studies A, B, and C was 916, 1713, and 3172, respectively (Figure 3b). Studies A and B shared 106 proteins, A and C shared 229, and B and C shared 402. All studies shared 45 proteins, namely GALNT7, PRKDC, GCC2, CXADR, INTS14, TFF3, MIEN1, SEC23B, CASP6, ICAM1, MYDGF, ARHGEF2, NSFL1C, ASNS, MUC5AC, UBR4, EGFR, COPA, ALDH3A1, PSAT1, SRM, EEFSEC, PGRMC1, CGNL1, COPB1, IVNS1ABP, LYPLA1, GOLGA4, NAA15, IDH2, LAMB2, JCHAIN, EIF2S3, S100A13, SURF4, MYOF, SH3GL1, FKBP10, KIAA1217, CPA3, IFI44L, PITRM1, ESRP1, CSNK1A1, and UBA6 (Supplementary Materials). A total of 10 proteins identified as regulated were highlighted as the most reported in the studies included in this analysis: EGFR, PGRMC1, CSE1L, MYDGF, STMN1 and CASP3 were regulated in four studies, and ANXA2, YBX1, ANXA1, and MYH11 were regulated in three studies (Figure 3c).

## 4. Discussion

The present manuscript brings together the available evidence on the proteomic profile of endometrial cancer, namely through identifying the differentially expressed proteins in several types of samples, emphasizing their potential as biomarkers for this gynecologic malignancy. The studies included in this review report the identification of hundreds or even thousands of proteins using different proteomics-based methodologies, which may indicate that these techniques can, in fact, help to search for new disease markers.

An early diagnosis of endometrial cancer not only leads to a better prognosis but also significantly impacts the survival rate of these patients. The timeliness of the diagnosis may even allow for a conservative approach for young women and patients who cannot undergo surgical treatment [75]. Among the different types of samples used, endometrial tissue and serum were preferentially selected for biomarkers search, resulting in the identification of a large number of differentially expressed proteins. Beyond that, some of these proteins have been mostly proposed as biomarkers for an early diagnosis, tailored treatment and prognostic evaluation of endometrial cancer.

Of the proteins identified in endometrial tissue, the most representative sample, the ANXA family, ENO1/ENOA, HSP family, S100A family, and PKM isoforms stand out as some of the most widely identified throughout different studies. These proteins demonstrate promising performance in detecting endometrial cancer, distinguishing tumors from normal samples, even from hyperplasia, at different stages, grades, and subtypes; as prognostic markers, recognizing primary from metastatic tumors, and predicting endometrial cancer risk. The annexin A protein family (ANXA) is a set of proteins with a recognized role in disease evolution, spreading, invasion and metastatic process [76]. As the most studied member of this family, ANXA2 is considered a possible cancer biomarker for several malignancies [77,78,79,80], including endometrial cancer [78]. Likewise, ANXA1 is associated with cancer; however, its role in proliferation and metastasis is controversial [81]. More recently, the ANXA1 expression in cancer was associated with the disease progression by mediating signaling pathways [82]. Moreover, its connection with the tumor microenvironment and cancer cells must be explored as an anticancer therapeutic target [83]. In colon cancer, ANXA1 was considered a possible druggable target [84]. The alpha-enolase (ENO1/ENOA) was another protein proposed as a diagnosis and prognosis biomarker for endometrial cancer. This protein plays a relevant role in various hallmarks of cancer, being involved in cancer development, invasion and resistance to therapy [85]. In several malignancies, i.e., bladder, breast, colorectal, lung, and gastric cancer, the ENO1 expression was associated with a worse prognosis [86,87,88,89,90]. In endometrial cancer, two members of the heat shock proteins (HSP) family, HSPA5 and HSPA8, were proposed as good markers for diagnosis and prognosis [34,43,54]. In bladder cancer and lung squamous cell carcinoma, the expression of HSPA5 was associated with disease development and prognosis [91,92,93]. In the same direction, an overexpression of HSP8 was found in triple-negative breast cancer and correlated with a poor prognosis [94]. Also, in acute myeloid leukemia, HSP8 was considered a prognosis biomarker [95]. In endometrial cancer, HSPA8 was also indicated as a potential therapeutic target [34].

Another family of proteins extensively considered as candidates for diagnosis and prognosis of endometrial cancer is S100A. S100A proteins are calcium-binding proteins involved in carcinogenesis and disease progression [96], including breast cancer, lung cancer, and melanoma [97], with a prognostic value in ovarian and breast cancer [98,99]. Moreover, this family of proteins is also responsible for drug resistance in a wide list of tumors, including endometrial cancer [100]. A differential expression of pyruvate kinase muscle (PKM) isoforms is also present in cancer. While PKM1 was associated with resistance to therapy [101], PKM isoform 2 is considered responsible for cancer growth [102]. Moreover, PKM2 has also been explored as a possible cancer-detection marker, associated with carcinogenesis [103].

Several blood-based protein biomarker candidates for endometrial cancer detection were suggested, including the Apolipoprotein family (APOA1/4, APOC1/2/3, and APOE), Clusterin (CLU), Inter-alpha-trypsin inhibitor heavy chain (ITIH2 and ITIH4), and Antithrombin III (ATR/SERPINC1).

Metabolic dysregulation is known to be one of the implicated mechanisms in endometrial cancer development [104]. Apolipoproteins are implied in lipid metabolism, and their differential serum expression after an 8 h fasting period suggests a systemic impairment of lipid metabolism in endometrial cancer and endometrial hyperplasia patients [104]. APOA1 is a major lipoprotein found in high-density lipoproteins (HDL) and possesses significant anti-inflammatory and antioxidant properties [105]. The role of inflammation as a crucial component in tumor progression is well recognized, underscoring the importance of this protein in various cancers. A downregulation of APOA1 has been observed in the serum of patients with several malignancies, indicating unfavorable prognosis, including ovarian, breast, and pancreatic cancers [106,107,108]. Conversely, an increased expression of APOA1 has been seen in other types of cancers, such as small-cell lung carcinoma, hepatocellular carcinoma, and bladder cancer [109,110,111]. In breast cancer, the role of APOA1 has been controversial [107,112,113]. In our review, three studies demonstrated that APOA1 expression was inversely associated with endometrial cancer [58,59,60], while one study found that higher APOA1 expression was positively associated with the disease [62]. However, the methodologies differed between these studies, as well as the sample types. In the latter study, only exosomes from the serum were analysed, rather than the whole serum [62]. Further research with larger sample sizes and standardized methodologies is needed to clarify these findings. An overexpression of APOC2 and APOC3 was reported by some studies reviewed in this manuscript and has been investigated in other cancers. Although APOC2 has been implied as a biomarker in pancreatic and cervical cancer [114], an overexpression of APOC3 has been described in ovarian cancer and in the recurrent disease of small-cell lung cancer patients [115]. Concerning APOA4, several proteomics studies have identified low levels, which were associated with various forms of cancer, including epithelial ovarian, hepatocellular, pancreatic, oral and papillary thyroid carcinoma [109,116,117,118]. In recent years, APOE has frequently appeared in tumor research and has gradually become recognized as a tumor biomarker. This protein plays a key role in tumorigenesis and progression, including cell proliferation, angiogenesis, and metastasis. Its overexpression has been reported in various cancers, such as gastric, lung, prostate, thyroid, ovarian, breast cancer, and glioblastoma [114,119,120,121,122,123,124].

In our review, two studies described the upregulation of APOE in endometrial cancer patients and its precursors [57,62], while one study found it to be downregulated [59]. To the best of our knowledge, this is the only study that reported an association between APOE and cancer. However, the downregulation was not further validated, and the results should be interpreted with caution due to the small sample size (10 endometrial cancer patients and 10 controls). Further research with larger sample sizes and standardized methodologies is needed to clarify these findings. Known to be involved in the clearance of cellular debris and apoptosis, CLU is an extracellular chaperone associated with tumor progression in multiple malignancies such as bladder, colon, hepatocellular carcinoma and renal cell carcinoma [125,126], and resistance to radiotherapy [127]. Regarding inter-alpha-trypsin inhibitors heavy chain [ITIH], ITHI4 is an acute-phase plasma glycoprotein produced by the liver and released into the bloodstream [128]. Currently, ITIH4 is thought to play a significant role in the genesis, development, invasion, and metastasis of various solid tumors. It has also been investigated as a potential biomarker in hepatocellular and gastric carcinomas [128,129]. SERPINC1 is an important serine protease inhibitor which has also been investigated as a biomarker in other malignancies, including hepatocellular carcinoma [130] and central nervous system lymphomas [131].

From our analysis of eight studies [21,36,39,40,47,49,52,66] to evaluate the uniformity of the potential biomarkers identified, ten proteins stood out as the most reported in the studies included in this manuscript. From these, EGFR, PGRMC1, CSE1L, MYDGF, STMN1 and CASP3 were reported in four studies. The epidermal growth factor receptor (EGFR), which regulates epithelial tissue development and homeostasis, has been implicated in tumorigenesis in several types of cancer [132]. In endometrial cancer, EGFR has been shown to be activated, leading to endometrial cancer progression [133]. Another identified protein was membrane-associated progesterone receptor component 1 (PGRMC1), a heme-binding protein implicated in several cellular functions. PGRMC1 is increased in ovarian and endometrial cancers, and this increase contributes to tumor progression. Although the clear mechanism is not yet well understood, it is known that PGRMCs play a significant role in survival pathways that attenuate stress-induced cell death [134].

The exportin-2 (CSE1L) protein is highly expressed in cancer and regulates invasion and metastasis of cancer cells, being highly related to high cancer stage, high cancer grade, and worse outcomes of patients [135,136]. This was a protein which was also detected in our analysis. Myeloid-derived growth factor (MYDGF), a protein involved in the protection and repair of the heart after myocardial infarction [137] was also identified in our analysis. MYDGF is overexpressed in different types of cancer cells and silencing it has been shown to decrease cancer cell proliferation [138,139]. Stathmin (STMN1) is a structural microtubule-associated protein that binds to microtubule protein dimers, destabilizing the microtubules. In cancer, increased expression of this protein was related to poor survival and a high risk of metastasizing [140]. Finally, caspase-3 (CASP3), a protein associated with cell apoptosis, also came up in our analysis. The role of CASP3 in cancer has been widely discussed in the scientific community. CASP3 is shown to be downregulated in several types of cancer; thus, activating it might serve as a way to kill cancer cells and improve survival [141]. The analysis of potential biomarkers for endometrial cancer is crucial to allow early detection and improve patient outcomes. These identified biomarkers can provide valuable insights into the molecular mechanism underlying cancer development and progression, contributing to a more precise diagnosis and prognosis. This can also help develop targeted therapies, minimizing the need for invasive procedures and increasing the chances of therapeutic success.

Although the identification of potential markers for endometrial cancer can be consensual within the same type of sample, and many of the proteins identified in the proteomic profile of endometrial cancer using tissue and serum are transversal to the various studies, different samples appear to provide different information. On the one hand, these findings may reinforce the usefulness of using clinical samples in biomarker research, particularly if they provide a specific proteomic signature. On the other hand, they may indicate that the best way to identify these molecular markers may involve combining several types of samples from the same individual for an accurate and timely diagnosis and useful in prognostic stratification.

Other types of clinical samples, such as cervicovaginal fluid and urine, as well as human endometrial cancer cell lines, were used in the articles reported in this review; however, there was no convergence regarding the biomarkers suggested for endometrial cancer. One limitation of this review that should be noted is that the number of studies reporting the use of the abovementioned samples is substantially lower than those using endometrial tissue and serum, which may explain these findings. Moreover, in general, the number of samples per group of study and the proteomic technique employed should also be taken into consideration in the analysis and interpretation of these results, given that a great deal of variability was observed between the included studies.

Globally, the studies examined in this manuscript emphasized encouraging findings. However, there appears to be a gap in the discovery of predictive biomarkers of response to therapy or even the discovery of new therapeutic targets. Although peri- and postmenopausal women are the most affected by endometrial cancer, approximately 14% of patients are under 50 years old [142], being a challenge in terms of diagnosis and treatment [143]. These young women, who can face limitations regarding conservative therapeutic options, could benefit from a fertility-sparing minimally invasive therapy for endometrial cancer. Thus, further studies are demanding to identify and explore the usefulness of molecular markers as possible therapeutic targets for endometrial cancer.

## 5. Conclusions

Proteomics-based approaches seem to be a valuable tool for the identification of cancer biomarkers. Research using clinical samples, namely endometrial tissue and serum, pointed out candidates for detection and prognosis biomarkers, revealing them to be reliable sources of proteins. However, there is a lack of studies exploring novel molecular therapeutic targets for endometrial cancer. 

## Figures and Tables

**Table 2:** Studies using serum and plasma samples.

Ref.	Sample Number	Age	Normal Samples	Pathological Samples	Methodology	Upregulated Proteins	Downregulated Proteins	Validation
[55]	25	35–65 years	Normal Endometrium (*n =* 13)	Endometrial cancer (*n =* 12)	2D-DIGE and MALDI-TOF	ABCG, ATR, CLU, LRG1	SERPINA1, KNG1	ELISA
[56]	70	ND	Normal Endometrium (*n =* 30)	Endometrial cancer (*n =* 40)	2DICAL and nano-LC-MS/MS	C4A, C3	APOA4	Immuno-blotting
[57]	27	ND	Normal Endometrium (*n =* 7)	Endometrial hyperplasia (simple *n =* 6, complex *n =* 4), atypical (*n =* 4)) Endometrial carcinoma (*n =* 6)	iTRAQ and 2D LC-MS/MS	SAA1, SAA2, APOC2, APOE	APOA4, ITIH4, HRG	NA
[58]	30	36–48 years	Normal Endometrium (*n =* 15)	Endometrial cancer (*n =* 15)	2D-DIGE and LC-MS/MS	CLU, SERPINC1, ITIH4, C1R, APOC3, DSC1	APCS, C9, APOA1, ALB, ITIH2, APOA4, CFHR1, ACTB	WB
[59]	20	ND	Normal Endometrium (*n =* 10)	Endometrial cancer (*n =* 10)	2D-DIGE and LC-MS/MS	APOC3, APOC2, SERPINC1, C1R, SERPINA1, A2M, CLU	APOA1, APCS, APOE, CD5L, CFHR1, VTN, C9, C8A, ALB, C4BPA, IGHM, ITIH2, FLG2, SBSN, APOA4, CPS1	WB
[60]	105	ND	Normal Endometrium (*n =* 40)	Endometrial cancer (*n =* 65)	SELDI-TOF MS	APOC1	APOA1	Cohort
[61]	12	33–68 years	Normal Endometrium (*n =* 4)	Endometrial cancer (*n =* 8)	LC-MS/MS	FAM83D	NA	WB
[62]	72	48–88 years	Normal Endometrium (*n =* 36)	Endometrial cancer (*n =* 36)	LFQ-MS	APOA1, HBB, CAH1, HBD, LPA, SAA4, PF4V1, APOE	IGLV3–19, IGKV3–20	WB
[63]	16	43–58 years	Normal Endometrium (*n =* 8)	Endometrial cancer (*n =* 8)	1D-GE and nano-LC- MS/MS and Q-TOF-MS/MS and FT-ICR- MS/MS	FRAS1	NA	WB
[64]	160	32–80 years	Normal Endometrium (*n =* 120)	Endometrial cancer (*n =* 40)	HPLC-ESI-MS	NA	NA	Cohort

Abbreviations: ND, Not Defined; NA, Not Applicable.

**Table 3:** Studies using cervicovaginal fluid samples.

Ref.	Sample Number	Age	Normal Samples	Pathological Samples	Methodology	Upregulated Proteins	Downregulated Proteins	Validation
[65]	19	N—50–81 years EC—52–84 years AH—57–78 years	Normal Endometrium (*n =* 7)	Endometrial Cancer (*n =* 9), Atypical Hyperplasia (*n =* 3)	HPLC-MS/MS	HSP10, HSP60, HSP71, HSP75, S100A8, S100A9, SCP2, PK-M1/M2, PGAM1, ENO1, SERPINA1	NA	Transcriptomics
[66]	45	23–93 years	Normal Endometrium (*n =* 21)	Endometrial Cancer (*n =* 24)	nano-UHPLC and Tims-TOF MS and LC-PRM	LDHA, ENOA, PKM, SERPHINH1, VIM, CSE1L, TAGLN, PPIA	NA	NA

Abbreviations: NA, Not Applicable.

**Table 4:** Studies with urine samples.

Ref.	Sample Number	Age	Normal Samples	Pathological Samples	Methodology	Upregulated Proteins	Downregulated Proteins	Validation
[67]	18	Age matched	Normal Endometrium (*n =* 11)	Endometrial Cancer (*n =* 7)	MALDI- TOF and LC-MS/MS	AZGP1, MPG	CD59	NA
[68]	12	55 years	Normal Endometrium (*n =* 7)	Endometrial Cancer (*n =* 5)	HPLC-ESI-MS/MS	NA	HSPG2, VTN, CDH1	NA
[69]	104	52–73 years	Normal Endometrium (*n =* 50)	Endometrial Cancer (*n =* 54)	SWATH- MS	SPRR1B, CRNN, CALML3, TXN, FABP5, C1RL, MMP9, ECML1, S100A7, CFI	NA	NA

Abbreviations: NA, Not Applicable.

**Table 5:** Studies using endometrial cancer cell lines.

Ref.	Disease, Model	Methodology	Upregulated Proteins	Downregulated Proteins	Validation
[70]	Endometrial Cancer, KLE and HEC1	2D LC-MS/MS	CPN10, PK-M1/M2, S100A11, IGFBP2/3/4/6/7, PLK1, SERPINA1, MIF	NA	Cohort
[71]	Endometrial Cancer, HEC1A and IK	iTRAQ, nano LC-MS/MS	BST2	NA	IHC
[72]	Endometrial Cancer, 2D vs. 3D, Human	TMT-HPLC and LC-MS/MS	HK2, PFKFB3, GPRC5A, HIF pathway	NA	PCR and WB
[73]	Endometrial Cancer, 2D vs. 3D, Human	2D-DIGE and MALDI- TOF-MS	VDAC1, ANXA4, PHB1	HSP8, VIM, TUBB, ENO1, AHCY, PGK1, ALDOA, LDHB, PSME2, PRDX6, PRDX1	WB
[74]	Endometrial Cancer, Co-Culture, Human	LC-MS/MS and IPA Analysis	ARPC2, PPP1R12A, ARPC3, MSN, MAPK1, GRB2, EIF2AK2, EIF2S2	NA	NA
[65]	Endometrial Cancer, Human	HPLC- MS/MS	NA	NA	Cohort

Abbreviations: NA, Not Applicable.

## Data Availability

Data obtained throughout this study is available in this manuscript.

## Supplementary Materials

The following supporting information can be downloaded at: https://www.mdpi.com/article/10.3390/biology13080584/s1, Table S1. Identification of common proteins among the three most correlated studies.
